# ZnO Nanowire Application in Chemoresistive Sensing: A Review

**DOI:** 10.3390/nano7110381

**Published:** 2017-11-09

**Authors:** Simas Rackauskas, Nadia Barbero, Claudia Barolo, Guido Viscardi

**Affiliations:** Department of Chemistry, NIS Interdepartmental Centre and INSTM Reference Centre, University of Turin, Via Pietro Giuria 7, 10125 Turin, Italy; nadia.barbero@unito.it (N.B.); claudia.barolo@unito.it (C.B.); guido.viscardi@unito.it (G.V.)

**Keywords:** ZnO, nanowire, sensors, chemoresistive, biosensing

## Abstract

This article provides an overview of the recent development of ZnO nanowires (NWs) for chemoresistive sensing. Working mechanisms of chemoresistive sensors are unified for gas, ultraviolet (UV) and bio sensor types: single nanowire and nanowire junction sensors are described, giving the overview for a simple sensor manufacture by multiple nanowire junctions. ZnO NW surface functionalization is discussed, and how this effects the sensing is explained. Further, novel approaches for sensing, using ZnO NW functionalization with other materials such as metal nanoparticles or heterojunctions, are explained, and limiting factors and possible improvements are discussed. The review concludes with the insights and recommendations for the future improvement of the ZnO NW chemoresistive sensing.

## 1. Introduction

ZnO nanowires (NWs) have attracted a great deal of interest due to their exceptional properties. ZnO NWs, because of their biocompatibility, piezoelectricity, and optoelectronic properties, are applicable for various electronic, photonic, biological, and energy related applications [1,2,3]. The most attractive semiconducting properties of ZnO are wide bandgap (3.37 eV), exciton binding energy (60 meV), high refractive index which is higher than 2 and also many other features of ZnO, including manufacturing techniques for high surface area, feasibility for wet chemical etching, structural stability and resistance to high-energy radiation. Moreover, ZnO has a tendency to form numerous nanostructures, because of the several fast grow directions available, which brings a diversity of methods for ZnO nanostructure synthesis, including by low-cost and low-temperature techniques. The potential is seen for application ultraviolet lasers [4], photodetectors [5], dye-sensitized solar cells [6,7], light-emitting diodes [8], and biomedical applications [9].

The names of ZnO nanomaterials such as nanowires [10] nanorods [11] or nanobelts [12] are used to characterize the shape of the obtained 1D material. ZnO NWs could be also grown in more sophisticated forms such as nanoforests [13], tetrapods [14,15,16,17], hierarchical nanowires [18], and many others [19,20,21] by simply controlling the crystal grow direction. ZnO nanowires are mostly grown by hydrothermal [22,23] or vapor transport methods [24,25]. A direct growth of ZnO NW on Zn metal is also attractive for its simplicity [26,27]. The variety of growth methods and availability of ZnO nanostructures gives a certain freedom to optimize ZnO NW sensors for the best surface reactivity and sensitivity.

ZnO NW application for sensing shows multiple advantages; especially interesting is a high surface ratio which provides an enhancement of the surface effects. Moreover, ZnO NWs are single crystals with a well-defined lattice, guiding to controlled reactions and providing stability. Comparing to polycrystalline structures, ZnO NW response is typically faster due to the reaction on the NW surface and no need for inter-grain diffusion. 

For the purpose of higher sensitivity and selectivity, ZnO NW sensor surface can be further modified or alternatively heterojunctions with other materials can be formed. Other forms of modification are NW morphology with the contact types or developing ZnO NW hybrid structures. Moreover, ZnO NW sensors can bring new functionalities with their flexible or stretchable configuration. 

## 2. ZnO Nanowire Sensors

The main principle of sensing is to get a measurable response (mostly electrical) from the added substance. High sensitivity (response to extremely small amounts), selectivity (ability to differentiate between various substances) and linearity (response is linear amount of substance measured, or response can be described by other simple law) are the main parameters for efficient sensors. Additionally, there are practical requirements such as stability, simplicity of manufacture and measurement, low cost, biocompatibility and many others, which depend on the application field.

Recently, sensors which work on the change of electrical parameters (chemoresistive, electrochemical, bio-electrochemical) have drawn much attention, due to their multiple advantages, such as simple measurement and manufacturing, low cost, diverse functionalization possibilities just to name few. This class of sensors, employing ZnO as a base material, will be covered in this review.

In a broad sense, ZnO NW resistive sensors for UV, gas and bio sensing have the same working principle: sensor response is related to the charge accumulated or transferred to NW. The working principle can be explained from the oxygen adsorption and desorption from the surface in gas sensors [28]. The mechanism is also attributed to CuO [29], SnO_2_ [16,30] and other metal oxides [31]. 

**Figure 1:**
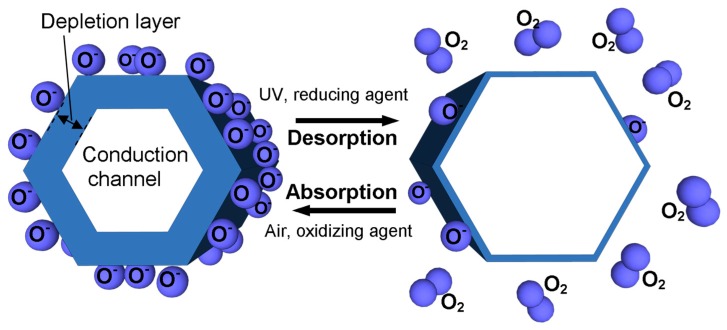
Schematics of unified ZnO nanowire (NW) chemoresistive sensing principle, based on the depletion layer width change with absorption-desorption of oxygen.

Most ZnO NW sensors are n-type semiconductors, since ZnO is an intrinsically stable electron-dominated conductor [32]; it is challenging to incorporate acceptors for stable conduction properties, therefore *p*-type ZnO NWs are rare [33]. Considering the *n*-type case, when ZnO NWs are exposed to atmosphere, oxygen adsorbs on the surface and becomes negatively charged (O^−^, O^2−^, or O_2_^−^) and extracts electrons from ZnO. The surface region that loses electrons is defined as the surface charge depletion layer (Figure 1). The depletion layer controls the effective conduction channel and increases the energy barrier height of the contact to NW or NW-NW junctions. When exposed to reducing gas, such as H_2_S, CO, and NH_3_, adsorbed O_2_ reacts with these gases and releases electrons back to the ZnO, which increases the electrical conductivity. As the depletion layer thickness is related to the oxygen coverage, the presence of reducing gas can diminish the thickness of the depletion layer, which increases the overall conductivity of the ZnO NWs. In the same manner, the oxidizing gases such as NO_2_ and O_3_ generally decrease conductance by extracting more electrons from the surface and increasing the depletion layer width [28,34]. Similarly, as in gas sensors, UV sensor response is associated with the depletion layer width. Illuminating ZnO NW with a wavelength, higher than the bandgap, photogenerated holes combine with the negative O_2_ ions, inducing desorption and increasing the conductivity. In biosensors, typically the ZnO NW surface is covered with a material (e.g., enzyme) which attaches the targeted substance from the analyte; consequently, charge is transferred to ZnO NW, changing its depletion layer width. However, in the case of biosensors, attention is paid to the immobilization of biomolecules on the surface, as is described in Section 2.2. 

**Figure 2:**
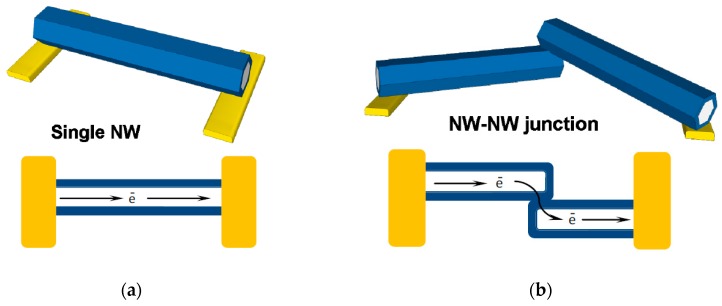
Schematics of ZnO NW sensor geometry types: (**a**) single NW; (**b**) NW-NW junction.

According to the device structure and working principle, nanowire sensors can be divided into two types: single nanowire connection and nanowire-nanowire junctions (Figure 2). Single connection devices rely on the response of a single NW, deposited or aligned between two metal contacts. Ohmic contacts are mostly used to contact ZnO NW; however, it is also possible to make Schottky contacts (e.g., contacting with Pt) in order to obtain a rectifying character or conduction [35]. However, most of research is done with NW-NW junction type of contacts, which is used not only because of the ease of sensor manufacture, but also due to the ability to control the barrier height. NW-NW junction sensors are typically more sensitive to small concentration changes, and have high rates of response as the conduction path involves tunneling through the depletion layer (Figure 2) [15]; however, in principle, single NW sensors can cover higher concentration ranges. Moreover, NW-NW junction integration into gas sensing devices is much more simple, as it relies on the randomly built conduction path between multiple NWs [15], whereas for the single NW sensors at least some degree of orientation is needed, wherefore ability to manipulate the NW or contacts is preferred [36]. 

Selectivity is another important feature of the ZnO NW sensors, which is connected to the ability of the sensor to discriminate among different types of chemical species. While, in biosensors, different analytes can be targeted with a special biomolecule, it is an especially difficult task to differentiate among the same type of gases (oxidizing or reducing), since depletion layer width change and consequently response will be in the same direction. Selective recognition of the gases can be addressed by variations in chemical adsorption and dissociation of the target gases at the NW surface; therefore, NW sensor selectivity can be approached by several methods: NW geometry control [36,37], NW functionalization [38,39], selective contact formation [40], heterojunction [41], operating temperature modulation [42], and sensor array formation [43].

### 2.1. ZnO NW Heterojunction

Heterojunction formation on ZnO NW, or simply a junction with other material, is mainly targeted to improve response and selectivity. A common choice for heterojunction is the use of metal oxides such as SnO_2_, because of high sensitivity. It was shown that ZnO NWs uniformly covered with the outer layer of *n*-type SnO_2_ nanoparticles [41] considerably improved the sensor response. Comparison of the gas sensor performance between SnO_2_, ZnO NW and the formed SnO_2_/ZnO NW heterojunction showed that, after functionalization, SnO_2_/ZnO NW sensor was responding at a high rate, selectivity and repeatability to *n*-butylamine gas was good, and therefore it can be applied for organic amine sensors. Another study used SnO_2_ NWs growth on ZnO NW for volatile organic compound sensing [44]. Advantageous sensor performance comparing to the pure ZnO NWs was explained by potential barriers, which were forming at the SnO_2_/ZnO. Moreover, the geometry of SnO_2_ nanowires affects the selectivity of triethylamine, toluene, ethanol, acetic acid, acetone, and methanol, allowing ZnO/SnO_2_ heterostructures to discriminate acetone from other volatile compounds.

**Figure 3:**
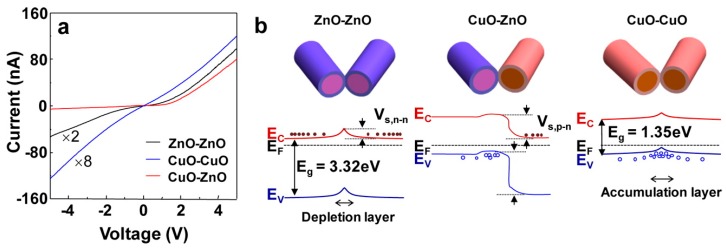
Comparison of contacts between ZnO and CuO NWs: (**a**) current-voltage (I-V) curves obtained at room temperature, demonstrating the characteristic nonlinear Schottky-like transport behavior; (**b**) Energy band diagram for every interconnection type between NWs. Reproduced with permission from [45]. Copyright American Chemical Society, 2013.

Another option is to use ZnO NW heterojunction with *p*-type materials, forming local p-n junctions. Park et al. studied ZnO NW junction with CuO NW, by growing long crystalline nanowires and forming air-bridge-structured junction [45]. It was shown that formation of nanoscale *p*-*n* junctions lowers sensor conductance by two orders of magnitude (Figure 3). The current-voltage I-V characteristics of both ZnO NW and CuO NW contacts were symmetric, while ZnO/CuO NW heterocontacts were asymmetric, which indicate the built-in potential established near the *p*-*n* junction. *P*-type material plays the role of catalyst and expands the adjacent electron depletion layer of ZnO NWs. However, sensitivity toward H_2_, CO and NO_2_ gases was lower of such *p*-*n* heterocontacts than *n*-*n* contact between ZnO NWs.

Zhang et al. reported an increased sensitivity of ZnO NW with co-precipitated *p*-type CuO flower-type structure for ethanol vapor sensors [46]. The ZnO/uO heterojunction demonstrates 2.5 times higher ethanol response at 300 °C compared to ZnO NWs without CuO. Good selectivity, long-term stability and also response and recovery time of 7 and 9 s, respectively, were demonstrated. The improved ethanol response was also due to a widening of depletion layer on the ZnO/CuO interface.

In order to make a *p*-*n* heterojunction with ZnO NWs it is also possible to use other *p* type metal oxides, such as NiO [47,48], Cr_2_O_3_ [49,50], Mn_3_O_4_ [51] or modify with *p*-typematerials, such as cobalt phthalocyanine (CoPc) [52].

UV sensor performance can also be enhanced applying ZnO NW heterojunctions with *p* type oxides such as CuO [53] or NiO [54]. Ultrafast response to UV in µs range was obtained at ZnO NW contact with *p*-Si [55], which was explained by pyro-phototronic effect [56]. The pyro-phototronic results are from three elements: pyroelectric effect, photonic excitations, and semiconductor properties, the coupling of which paves a way to reach ultrafast photosensing performance with optoelectronic processes. Moreover, the response time of ZnO NW UV sensors decreases with increasing illumination intensity; thus, this could be potentially applied for ultrafast detection of intensive light.

Another interesting option is to decorate ZnO with noble metal particles. Metal nanoparticles can be employed to improve optical absorbance or emission in semiconductors, due to a high plasmon interaction with electromagnetic fields. ZnO NW gas sensing of ethanol can be improved by adding noble metallic nanoparticles, for example Au, Pt [57] or Ag [58]. Alternatively, a selective Pd contact with ZnO NWs [40] can be used for various gases such as H_2_, CH_4_, H_2_S and CO_2_ at different operating temperatures, where high efficiency in hydrogen detection was reported. Moreover, Pt contacted ZnO NW networks can be used as self-powered UV sensors, since excellent photoresponse properties to 365 nm UV irradiation was obtained at zero bias [31].

#### Combination of UV and Gas Sensing in ZnO NW 

The high operating temperatures (typically about 350 °C) are essential for gas detection and sensing, which is a major technical limitation in applicability. Moreover, adsorption of water on the ZnO surface leads to a decrease in surface potential, at relative humidity higher than 14% due to adsorbed water molecules increasing the surface electron density [59]. Irradiation of ZnO by photons with an energy greater than the band gap (3.37 eV) changes adsorbed oxygen species on the surface, which is a practical alternative for achieving chemical reactions without the necessary heating. However, the UV activation shows an order of magnitude lower sensitivity compared to the same sensors activated by traditional heating methods [60]. UV activation, in order to increase the response, is used for ZnO films [59,61] and also for other materials [62,63]. A transparent ZnO NW sensor, which detects both UV and ethanol gas, was fabricated and deposited onto a silicon solar cell [64]. In UV sensing, the current rise was obtained in 137 s. In ethanol vapor detection, UV was used for sensitivity improvement. The ZnO NW sensor response increased by 13% with an ethanol gas concentration change of 100 ppm at 53 °C (heat generated by the c-Si solar cell). The sensor response is approximately zero without solar illumination.

**Figure 4:**
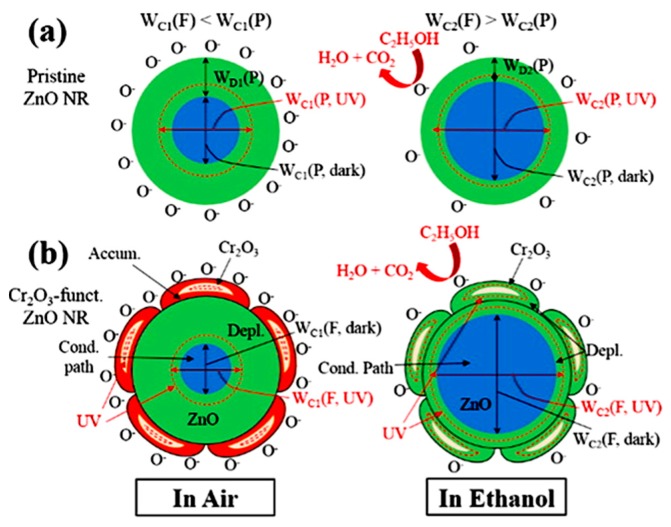
Schematic diagrams: (**a**) pristine ZnO NW; (**b**) Cr_2_O_3_-modified ZnO NW in the dark and under UV illumination in air and ethanol showing the depletion layer and conduction path. Reproduced with permission from [65]. Copyright American Chemical Society, 2016.

Combining the response to chemical substance with a UV illumination, it is possible to get a synergy of these two effects and an increase of the performance of ZnO NW sensors (Figure 4); however, there are still only some studies on the combination of more than two effects on the performance of NW sensors. Synergistic effects of Cr_2_O_3_ functionalized ZnO NW sensor with UV irradiation were demonstrated for the ethanol gas sensing [65]. The responses at room temperature to ethanol were increased by UV illumination by 3.8 times for the pristine and by 7.7 times for Cr_2_O_3_ functionalized ZnO NW sensors. Moreover, the Cr_2_O_3_ modified ZnO NW sensor demonstrated rapid response and recovery; moreover, selectivity was also increased. This shows that combining heterostructures with UV activation has a synergistic effect on sensor performance. The synergistic effects arise from the Cr_2_O_3_ catalytic oxidation of ethanol and also from conduction channel width change due to Cr_2_O_3_ nanoparticle effect on ethanol adsorption and desorption under UV illumination in the Cr_2_O_3_ modified ZnO NW sensor.

Similarly, gold-decorated ZnO thin films showed improved sensing properties compared to bare ZnO under UV illumination. The sensor showed a selective response to NO_2_ gas under green light illumination. Moreover, Au/ZnO sensor can detect SO_2_ gas in ppm level in humid conditions [66].

### 2.2. ZnO Nanowire Biosensors

**Figure 5:**
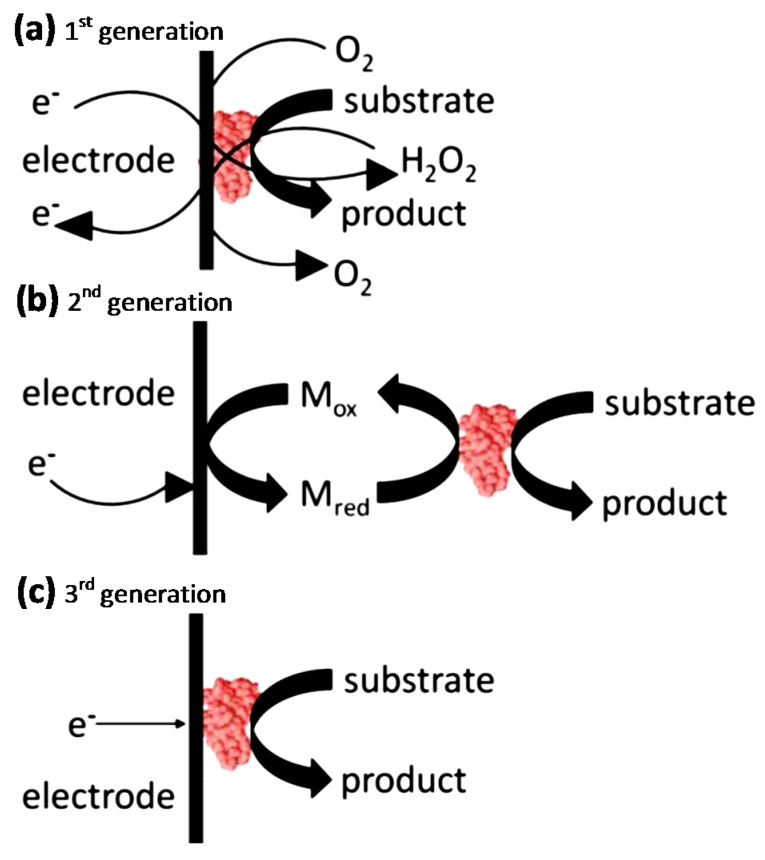
Schematic diagrams of three generations of sensors: (**a**) 1st generation; (**b**) 2nd generation and (**c**) 3rd generation [69].

We can identify three generations of biosensors [67]. In the first generation (Figure 5a), an electrical response is generated by the diffusion of the reaction products to the transducer. In the second generation (Figure 5b), instead, an initial redox reaction is performed by a mediator between the enzyme and its substrate. The enzyme and mediator are usually co-immobilized at an electrode surface in the third-generation of biosensors (Figure 5c); in this way, the biorecognition component is an integral part of the electrode transducer [68]. 

Electronic devices based on semiconductor NW have emerged as a potential platform for the qualitative and quantitative detection of chemical and biological species due to their ultralow detection limit, fast readout and easy fabrication [70].

In particular, ZnO possesses a series of properties and characteristics (high electron mobility, easiness of fabrication of ZnO, biocompatibility and low toxicity) that makes it a nice candidate for the construction of biosensors [71]. In particular, ZnO NWs, due to their low weight with extraordinary mechanical, electrical, thermal and multifunctional properties along with their high surface area, are suitable for adsorption or immobilization of various biomolecules such as proteins, enzymes or antibodies [9]. ZnO nanowires possess active surfaces that can be modified for the immobilization of a large number of biomolecules. These nanostructures can be perfect transducers for producing signals to interface with macroscopic instruments since they present diameters which are comparable to the sizes of the biological and chemical species being sensed [72]. Based on the specific feature of NW, biosensors may even go down to a single molecule detection [73].

Qualitative and quantitative detection of chemical and biological species is crucial in a huge variety of fields. If the literature on biosensors based on ZnO nanomaterials is quite rich, relatively few examples are focused on ZnO nanowires. Below, we will briefly summarize some recent findings reporting biosensors based on ZnO nanowires.

#### 2.2.1. Urea Biosensors

The importance of urea measurements in blood and serum is important for a certain number of diseases. ZnO nanomaterials comprising nanorods and nanowires have been used for the fabrication of urea biosensors [9]. Well-aligned ZnO NW arrays were fabricated on gold-coated plastic substrates by using a low-temperature aqueous chemical growth (ACG) method and were proved to be sensitive to urea detection at a concentration from 0.1 to 100 mM [74]. Urease was immobilized on the surface of the ZnO NWs using an electrostatic process. More recently, high quality ZnO NWs, synthesized by the chemical vapor deposition (CVD) method, were used for the fabrication of electrical biosensors based on field effect transistor (FET) for the simultaneously low and high concentrations detection of uric acid [73]. The obtained ZnO NW bioFET sensors could easily detect uric acid down to a concentration of 1 pM with 14.7 nS of conductance increase, and the response time turns out to be in the order of milliseconds.

#### 2.2.2. Glucose Biosensors

Glucose is a critical metabolite for living organisms, particularly for patients who are suffering from diabetes, and glucose sensors attracted a huge amount of interest being one of the most important sensing technologies in medical science, clinical diagnostics, and food industry [75].

High-density vertical ZnO NWs were synthesized using the vapor-phase deposition method on patterned Au/glass electrode substrate with and without Au nanoparticle (NP) modification. A huge enhancement of the sensitivity toward glucose was obtained with Au NP modification [76]. A similar approach has been followed for the fabrication of high density, well-aligned ZnO NWs decorated with Pt nanoparticles to fabricate the working electrode for a non-enzymatic glucose biosensor [77]. ZnO NWs were synthesized hydrothermally on a glass substrate. The Pt NPs decoration allowed to enhance the biosensor’s glucose sensitivity 10-fold in comparison with the pristine ZnO NWs electrode. The large specific surface area, abundant microspace, small channels, and high isoelectric point (IEP) fracture of ZnO enable effective fluid circulation and good biocompatibility boding well for immobilization of enzymes. These characteristics were exploited for the immobilization of glucose oxidase on a glucose enzymatic biosensor composed of ZnO NWs supported by silicon NWs (ZnO/Si NWs) [78]. A Si NWs/ZnO nanowire nanocomposites enzymatic biosensor exhibited very strong and sensitive amperometric responses to glucose, even in the presence of common interfering species, and showed a high sensitivity of 129 μA·mM^−1^, low detection limit of 12 μΜ, and good stability as well as reproducibility. Very recently, a roll-to-roll flexographic printing technique was used for the fabrication of a three electrode electrochemical enzymatic biosensor consisting of ZnO NWs [79]. This biosensor device showed a typical sensitivity of 1.2 ± 0.2 μA·mM^−1^·cm^−2^ with a linear response to the addition of glucose over a concentration range of 0.1 to 3.6 mM.

#### 2.2.3. DNA Detection

Nanomaterials can provide good opportunities for sequence-specific target DNA detection as a medium for signal amplification. The possibility of using ZnO NWs to fabricate electrochemical DNA biosensors was explored some years ago. In order to improve the sensitivity, multi-walled carbon nanotubes (MWCNTs) and gold nanoparticles (Au NPs) were employed. The resulting device was able to quantitatively detect target DNA from 1.0 × 10^−7^ to 1.0 × 10^−13^ M with a detection limit of 3.5 × 10^−14^ M [80]. Another ZnO NWs/Au electrode showed to be a suitable platform for the immobilization of DNA for the rapid detection of a sequence specific for the breast cancer 1 (BRCA1) gene [81]. This DNA biosensor has the ability to detect the target sequence in the range of concentration between 10.0 and 100.0 μM with a detection limit of 3.32 μM. A further sensitive and in situ selective label-free DNA sensor, based on a Schottky contacted ZnO NW device, has been fabricated. The performance of this device was significantly enhanced by the presence of piezotronic effect [82].

## 3. Conclusions

There is a growing interest in ZnO NW application for sensing due to their high temperature stability, biocompatibility, simple synthesis route and sensor manufacture. In this review, we have summarized recent strategies to enhance ZnO NW sensor performance. Several conclusions could be made with the proposal for future trends:

- Many synthetic routes for ZnO NWs are already found, and different ZnO NW structures can be obtained by varying growth conditions. Decoration with metal particles is easily achieved; however, there is still a lack of comparative studies where the sensor performance is related not to the synthesis conditions but to the morphology of the sensor, namely the size of a conductive channel and how it is influenced by absorbed material.
- Heterojunction brings another possibility for the improvement of the sensing which can be achieved with comparingly facile fabrication techniques. Junctions with other p or n materials can be made with controlled surface coverage and thickness; still, there is a need to optimize ZnO NW interface with other materials in order to obtain high efficiency sensing.
- Using UV activation for bio and chemoresistive sensors shows considerable improvement in sensing. However, there is still a lack of understanding for the interplay of several effects (UV, temperature, oxygen adsorption, etc.), especially at the junctions of NW to NW or *p*-*n* junctions.
- The unique properties of ZnO NWs, simple fabrication and the possibility for suitable surface functionalization of the NWs make them exemplar as biosensor materials for a great variety of applications.
- The devices can be fabricated by roll-to-roll printing, which is suitable for low-cost high-volume production and a spread of large-scale commercialization of the biosensors.
